# What Circumstances and Conditions Modify the Action of Solvents?

**Published:** 1891-03

**Authors:** F. W. Fleming


### What Circumstances and Conditions Modify the Action of Solvents?

BY F. W. FLEMING.

When a solvent acts upon a body it may either effect a simple solution or may enter into chemical combination with it, or with some constituent of it. In case of simple solution a purely physical action occurs, the dissolved body being unchanged in properties, and no manifestation of heat is observed, as would be the case in chemical action; on the contrary there is a reduction of temperature, such as is always noticed in the merely physical change from the solid to liquid form, or from the liquid to the gaseous. Potassic nitrate and ammonic chloride dissolved in water form a very efficient refrigerant in the absence of snow or ice. Again the action of the well-known Carre freezing machine is based on this absorption or disappearance of heat as the liquid ammonia gas rapidly passes under diminished pressure, from a liquid into a gaseous state. The machine itself being simply a contrivance which facilitates the conversion of the gas into a liquid under pressure, and the passage back again into a gas under diminished pressure.

A solution may be of varying strength, but usually for any given solvent there are limits. These limits are known as saturated solutions, either cold or hot, *e. g.* 100 c. c. of cold water (roughly speaking) will dissolve 20 grammes of ordinary nitre (potassic nitrate), if the water, however, be at a temperature of 100 degrees C. the 100 c. c. will take up about 140 grammes. Under certain conditions a further quantity may be dissolved, but it cannot be kept in solution by ordinary methods, it is then spoken of as a *supersaturated solution.* For example: boiling water will take into solution more than twice its weight of sodic sulphate. If this solution be allowed to cool in an open flask an abundant crystalization will take place, for cold water only dissolves about one-third its weight of this salt. If however, the flask be tightly corked whilst the solution is boiling, it may be kept indefinately without crystalizing. Upon removal of the cork the whole solution is seen to crystalize in a rapid and beautiful manner. This effect is probably due to the decrease in pressure brought about by corking the flask while the solution was boiling. The crystalization of super-saturated solution seems also to be affected by the contact of minute solid bodies (nuclei) derived from the air. This can be demonstrated as follows: Take a solution of sodic sulphate containing two-thirds its weight of the crystalized salt. While the solution is boiling close the flask by a cork, through which pass two short pieces of glass tube. Plug the tubes with cotton wool. After the solution becomes cold, if the cotton plugs are withdrawn, and one blows through either tube, crystalization does not take place. Apparently the air in its passage to the lungs and out again has lost these nuclei, for if air be blown from a bellows crystalization immediately occurs. Perhaps a still more striking proof is obtained by making a solution of alum, saturated at 149 degrees F. and allowing it to cool in a flask, the mouth of which is closed by a plug of cotton wool. It will remain as a clear solution as long as the cotton wool is left in the neck of the flask, and air only reaches it after being filtered by the wool. Once admit unfiltered air and crystalization commencing at a few points immediately underneath the opening of the neck progresses rapidly.

There are a number of curious phenomena connected with the dissolving of metals well worthy of study. One of the most striking is that known as the "passive state." Concentrated sulphuric and nitric acid do not act upon iron at the ordinary temperature, though when diluted they dissolve it readily. If a bar of iron be first immersed in strong nitric acid (sp. gr. 1.45) and then in dilute, the dilute acid will be unable to act upon it, in fact the iron has assumed the "passive state." It can be restored to the normal state, or prevented from assuming the passive state, by wiping it after taking it out of the strong nitric acid before immersing it in the dilute acid. The dilute acid will then quickly dissolve the iron. This action has been attributed to the formation of a coating of the magnetic oxide, which is sparingly soluble in strong nitric acid.

The chief methods adopted in the laboratory for bringing substances into solution are: pulverization of body, application of heat, and use of menstrunms that will either effect the required solution, as alcohol, chloroform, glycerine, etc., or that will enter into chemical combination with the body, as acids, alkalies, etc. If a body be not pulverized there is less surface exposed to the solvent, and in some cases a deposit may be formed which will stop further action. Heat as a rule hastens and cold retards solution. A notable exception to this rule is calcic oxide which is less soluble in boiling water than in cold.

After all, however, the main factor in solution is the menstrunm. Comparatively few bodies can be brought into simple solution, they must first be chemically charged. This is markedly true as regards the human organism. Some of our most important food stuffs are insoluble as we partake of them, but by the various secretions are chemically changed into soluble forms. Our nervous system controls these secreting organs, and hence must control solution. Of the nerves going to any given gland, one set governs the amount of transudation of water and salines which shall take place through them, and another controlling the production of these, so that the secreted fluid may be rich or poor in salines under different nervous conditions.

According to Dr. Miller, saliva in contact with amylaceous or saccharine foods, in a few hours gives rise to a strong acid reaction, due to generation of organic acids. Hence there must be in the human mouth a constant, though variable generation of acid, because of the impossibility of keeping the mouth perfectly free from foods, and from solutions of amyloids in saliva, which penetrate cracks, pits, and fissures, or are held by capillary attraction between the surfaces of the teeth in contact and there become acid by fermentation.

